# Consideration of Optimal Evaluation Metrics for Internal Gross Tumor Dose Relevant to Tumor Response in Multi-fraction Stereotactic Radiosurgery of Brain Metastasis

**DOI:** 10.7759/cureus.65338

**Published:** 2024-07-25

**Authors:** Kazuhiro Ohtakara, Kojiro Suzuki

**Affiliations:** 1 Department of Radiation Oncology, Kainan Hospital Aichi Prefectural Welfare Federation of Agricultural Cooperatives, Yatomi, JPN; 2 Department of Radiology, Aichi Medical University, Nagakute, JPN

**Keywords:** dose evaluation, maximal tumor response, local control, gross tumor volume, biologically effective dose, volumetric-modulated arc therapy, dose inhomogeneity, dose gradient, brain metastasis, stereotactic radiosurgery

## Abstract

Introduction

In stereotactic radiosurgery (SRS) for brain metastasis (BM), the target dose inhomogeneity remains highly variable among modalities, irradiation techniques, and facilities, which can affect tumor response during and after multi-fraction SRS. Volumetric-modulated arcs (VMAs) can provide a concentrically-layered steep dose increase inside a gross tumor volume (GTV) boundary compared to dynamic conformal arcs. This study was conducted to review the optimal evaluation method for the internal GTV doses relevant to maximal response and local control, specifically to examine the significance of the doses 2 mm and 4 mm inside the GTV boundary in VMA-based SRS.

Materials and methods

This was a planning study for the clinical scenario of a single BM and targeted 25 GTVs of >0.50 cc, including eight spherical models with diameters of 10-45 mm and 17 clinical BMs (GTV: 0.72-44.33 cc). SRS plans were generated for each GTV using VMA with a 5-mm leaf-width multileaf collimator and the optimization that prioritized the steepness of the dose gradient outside the GTV boundary without any internal dose constraints. The dose prescription and evaluation were based on the GTV *D*_V-0.01 cc_, a minimum dose of GTV minus 0.01 cc. Two planning systems were compared for the GTV - 2 mm and GTV - 4 mm structures that were generated by equally reducing 2 mm and 4 mm from the GTV surface. The *D*_eIIV_s, a minimum dose of the irradiated isodose volume equivalent to the GTV - 2 mm and GTV - 4 mm, were compared to other common metrics.

Results

The GTV - 2 mm and GTV - 4 mm volumes differed significantly between the systems. In the spherical GTVs, the irradiated isodose surfaces of GTV *D*_80%_ and *D*_50%_ corresponded to 0.4-1.6 mm (<2 mm) and 1.0-4.6 mm inside the GTV boundary, respectively. In the 25 GTVs, the GTV - 2 mm coverage with the *D*_eIIV_ varied from 83.7% to 98.2% (95-98% in 68% of the cases), while the GTV coverage with the GTV - 2 mm *D*_eIIV_ was 20.2-75.9%. In the 23 GTVs of ≥1.26 cc, the GTV coverage with the GTV - 4 mm *D*_eIIV_ varied from 1.9% to 55.6% (<50% in 87% of the cases). No significant difference was observed between the GTV *D*_50%_ and the GTV - 2 mm *D*_eIIV_, while the GTV - 4 mm *D*_eIIV_ was significantly higher than the GTV *D*_50%_. No significant correlations were observed between the GTV *D*_50%_ and the *D*_eIIV_s of the GTV - 2 mm and GTV - 4 mm.

Conclusions

The doses 2 mm and 4 mm inside a GTV have low correlations with the GTV *D*_50%_ and may be more relevant to maximal response and local control for SRS of BM. The *D*_eIIV_ instead of the minimum dose of a fixed % coverage (e.g. *D*_98%_) is suitable for reporting the doses 2 mm and 4 mm inside the GTV boundary in terms of avoiding the over- or under-coverage, with consideration to substantial variability in minus margin addition functions among planning systems. In VMA-based SRS with a steep dose gradient, the doses 2-4 mm inside a GTV decrease significantly as the GTV increases, which can attenuate the excessive dose exposure to the surrounding brain in a large BM due to the GTV shrinkage during multi-fraction SRS.

## Introduction

Stereotactic radiosurgery (SRS) is an indispensable therapeutic avenue for brain metastases (BMs), and its indication has been expanded to large (>3 cm) and/or multiple (>10) lesions, with the increasing leverage of frameless multi-fraction irradiation [1-3]. SRS has been performed in a variety of devices and techniques, in which the target dose heterogeneities range from homogeneous to extremely inhomogeneous, with significant variability among facilities [4,5]. Especially in multi-fraction SRS, even if the marginal dose of a gross tumor volume (GTV) is the same, the differences in the dose gradient inside the GTV boundary can lead to differences in the degree of tumor shrinkage, maximal response, and its durability during and after multi-fraction SRS [6,7]. Extremely inhomogeneous target dose and/or intentional internal dose escalation can contribute to excellent maximum response (reduction rate) and local control [8-15]. Specifically, the volume irradiated with ≥30 Gy (one fraction) in a GTV is large [8,10], and the GTV *D*_80%_, a minimum dose to cover 80% of a GTV, is high [12].

A GTV internal dose (dose inhomogeneity) was traditionally reported only in terms of the marginal dose and central (maximum) dose [5]. Subsequently, evaluation with the *D*_98%_ (or *D*_V-0.035 cc_ for <2 cc), *D*_50%_, and *D*_2%_ (or *D*_0.035 cc_ for <2 cc) has been recommended [16]. With the same equipment and irradiation technique, even if the GTV marginal dose and maximum dose are the same, the dose gradient several millimeters inward from the GTV boundary will vary considerably depending on the planning method [4,6,7]. Therefore, it is necessary to verify whether the conventional dose evaluation method ensures the optimal representative metrics to specify the differences in these internal dose characteristics.

Dynamic conformal arcs (DCAs) and volumetric-modulated arcs (VMAs) are frequently used irradiation techniques in linac-based SRS using a multi-leaf collimator (MLC) [17,18]. In DCAs, it is necessary to specify target dose heterogeneity such as 70% isodose covering in planning [4,17]. DCAs frequently require the use of dummy structures, a modified GTV as a surrogate for MLC adaptation, to improve dose conformity [19]. In addition, with the arcs arranged exclusively in the cranial hemisphere, the high-dose areas tend to be unevenly distributed toward the cranial side of a GTV [19]. In contrast, VMAs can provide extremely inhomogeneous GTV doses suitable for individual lesions through optimization that prioritizes the steepness of dose gradient outside a GTV boundary without any internal dose constraints or without fixing GTV dose heterogeneity [7,20]. VMAs can generate a concentrically-layered steep dose gradient inside the GTV boundary with excellent dose convergence toward the center, compared to DCAs [2,7,21].

In this study, we will consider the optimal dose evaluation method to appropriately specify the difference in dose gradient inward from a GTV boundary, between the GTV boundary and its center. Specifically, we will examine the significance of evaluating the doses 2 mm and 4 mm inside the GTV boundary and the appropriate metrics in VMA-based SRS for a single BM.

## Materials and methods

This was a planning study for the clinical scenarios of a single BM. The GTV for clinical BM was defined as in the previous study [20,22].

Table 1 shows the physical doses with the corresponding biologically effective doses based on the linear-quadratic formula with the alpha/beta ratio of 10 (BED_10_) being just over 80.00 Gy, 100.00 Gy, and 120.00 Gy in 1-10 fraction(s) [21].

**Table 1:** Physical doses with the BEDs 100 Gy and 120 Gy relative to a prescribed dose with the BED 80 Gy in 1-10 fractions. *The percentages of the physical doses with the BED of ≥100.00 Gy and ≥120.00 Gy, relative to the physical doses (100%) with the BED of ≥80.00 Gy in 1-10 fraction(s), in which those in 2, 7, and 9 fractions are excluded. The BED is based on the linear-quadratic formula with an alpha/beta ratio of 10 (BED_10_). The physical doses to second decimal places at which the BED_10_ is exactly 80.00 Gy, 100.00 Gy, and 120.00 Gy or more, respectively. BEDs: Biologically effective doses; fr: Fraction(s)

BED_10_	Fraction(s)	1 fr	3 fr	4 fr	5 fr	6 fr	8 fr	10 fr
BED_10_ 80 Gy	Physical dose (BED_10_)	23.73 (80.04)	36.24 (80.02)	40.00 (80.00)	43.01 (80.01)	45.50 (80.00)	49.45 (80.02)	52.47 (80.00)
BED_10_ 100 Gy	Physical dose (BED_10_)	27.02 (100.03)	41.79 (100.00)	46.34 (100.02)	50.00 (100.00)	53.07 (100.01)	57.98 (100.00)	61.81 (100.01)
	Relative dose* (%)	113.9%	115.3%	115.9%	116.3%	116.6%	117.2%	117.8%
BED_10_ 120 Gy	Physical dose (BED_10_)	30.00 (120.00)	46.85 (120.01)	52.11 (120.00)	56.40 (120.02)	60.00 (120.00)	65.83 (120.00)	70.42 (120.01)
	Relative dose* (%)	126.4%	129.3%	130.3%	131.1%	131.9%	133.1%	134.2%

Table 2 shows the BED_10_ of physical doses equivalent to 125% and 150% of a prescribed dose with the BED_10_ 80 Gy in 1-10 fraction(s).

**Table 2:** BED10 of the physical doses equivalent to 125% and 150% of a prescribed dose with the BED10 80 Gy in 1-10 fractions. Physical doses and the corresponding BED_10_ are displayed in second decimal places. fr: Fraction(s); BED_10_: Biologically effective dose based on the linear-quadratic formula with an alpha/beta ratio of 10

Relative dose	Fraction(s)	1 fr	3 fr	4 fr	5 fr	6 fr	8 fr	10 fr
125%	Physical dose (BED_10_)	29.66 (117.65)	45.30 (113.70)	50.00 (112.50)	53.76 (111.57)	56.88 (110.79)	61.81 (109.57)	65.59 (108.60)
150%	Physical dose (BED_10_)	35.60 (162.30)	54.36 (152.86)	60.00 (150.00)	64.52 (147.76)	68.25 (145.88)	74.18 (142.95)	78.71 (140.65)

The physical doses (%) of the BED_10_ 100 Gy and 120 Gy, relative to those of the BED_10_ 80 Gy (100%), definitely increase as the number of dose fractions increases (Table 1). In dose prescription with the BED_10_ 80 Gy to a GTV boundary with the same dose gradient inside the GTV, the internal BED_10_ steadily decreases with increasing the number of fractions (Table 2). Thus, the internal BED_10_ of a GTV decreases with increasing dose fractionation when the GTV dose inhomogeneity is equal.

Eight sphere structures with diameters ranging from 10 to 45 mm with a 5-mm increment were generated using a sphere drawing tool by MIM Maestro^®^ version 7.1.3 (MIM Software Inc., Cleveland, Ohio, USA) and were assumed as GTVs as in the previous study [7,22]. The head computed tomography (CT) images (voxel size 0.98 x 0.98 x 1 mm) and GTV localization (the right lateral thalamus) used were identical to those employed in the previous study [7,20,22]. Additionally, 17 clinical BMs of various volumes (>0.50 cc, 0.72-44.33 cc) were selected, and each original GTV was treated as a single lesion. Thus, a total of 25 GTVs were included in this study.

Each GTV was equally reduced by 2 mm and 4 mm using two planning systems, MIM Maestro and Monaco^®^ version 5.51.10 (Elekta AB, Stockholm, Sweden) by adding the same minus margin to the GTV, to generate the GTV - 2 mm and GTV - 4 mm structures, respectively [22]. The two types of GTV - 2 mm and GTV - 4 mm were compared, together with the calculated values for the sphere GTVs. The changes in these target volumes on the dose-volume histogram (DVH) in Monaco were also compared.

SRS plans were generated for each GTV. The dose prescription was based on the GTV *D*_V-0.01 cc_, a minimum dose to cover a GTV minus 0.01 cc [20]. The treatment platform was a 5-mm MLC Agility^®^ (Elekta AB, Stockholm, Sweden) mounted in a linac Infinity^®^ (Elekta AB, Stockholm, Sweden) with a flattening filter-free mode of a 6 MV X-ray beam, which provides a dose rate of up to 1400 monitor unit per minute [20,22]. Monaco was used to optimize VMA-based SRS [7,20,22]. The isocenter localization, arc arrangement, optimization method, and dose calculation algorithm were identical to those described in the previous study [20,22]. Following the completion of optimization, each prescribed dose was rescaled according to each coverage value (>95%) of the GTV *D*_V-0.01 cc_ [20].

The near-maximum dose of GTV was defined as *D*_0.01 cc_, and the GTV *D*_0.01 cc_ was expressed as a relative dose to the GTV *D*_V-0.01 cc_ (%) to represent the GTV dose inhomogeneity [20]. An irradiated isodose volume (IIV) of X Gy was defined as the total volume to which ≥X Gy was irradiated, including the GTV. A minimum dose to cover the IIV equivalent to a target volume on the DVH was defined as *D*_eIIV_ (eIIV: equivalent IIV), an alternative to the near-minimum dose of a non-GTV target volume [22].

For statistical analyses, paired nonparametric tests were used, considering the distributions of the variables. The Spearman’s rank correlation coefficient (SRCC) was used to evaluate the correlations between two numerical variables. The Wilcoxon signed-rank test (WSRT) was used to compare two numerical variables. Significance was considered at p < 0.05 (*), p < 0.01 (**), and p < 0.001 (***).

## Results

**Figure 1:**
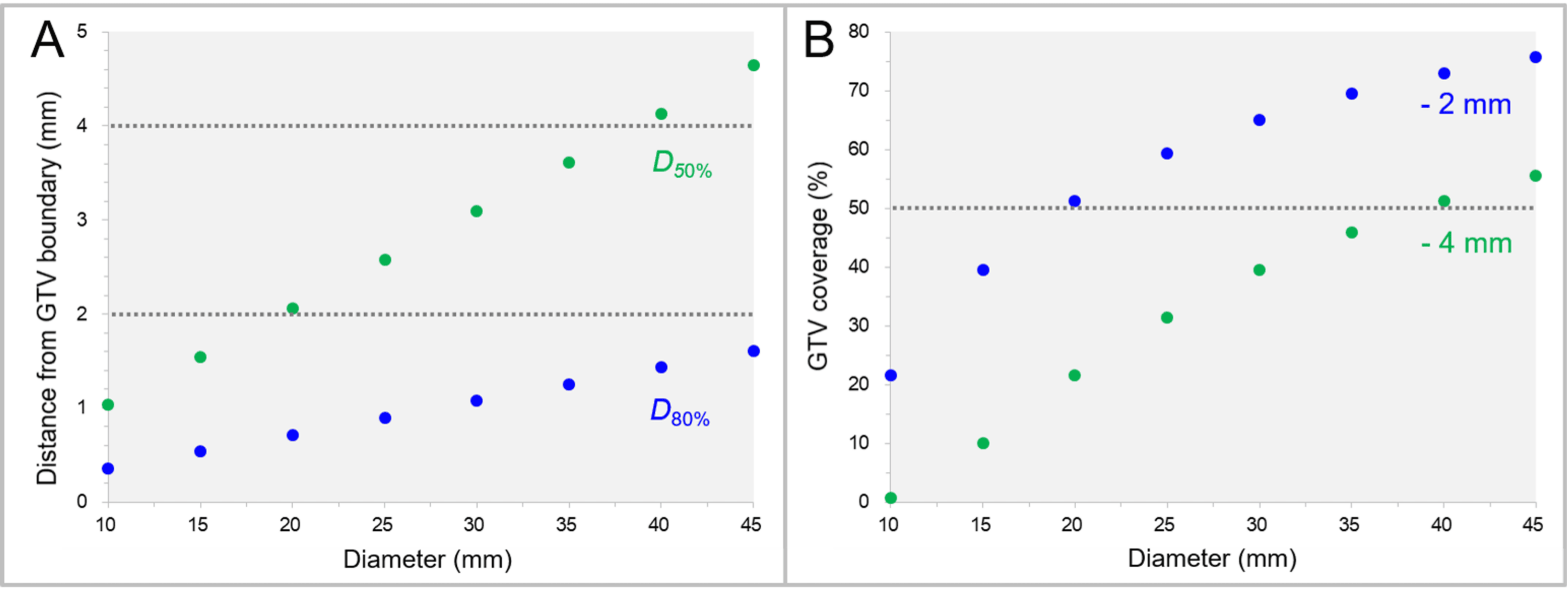
Distances of the irradiated isodose volume surfaces of spherical GTV D80% and D50% from the GTV boundary, and the GTV coverages by the 2 mm and 4 mm inner volumes of the GTV. The scatter plots show the correlations between the spherical GTV diameters and the distances of the irradiated isodose volume surfaces of the GTV *D*_80%_ and *D*_50%_ from the GTV boundary (A) and the GTV coverage values by the GTV - 2 mm and GTV - 4 mm structures (B). All the structure volumes are based on calculations. GTV: Gross tumor volume; *D*_X%_: A minimum dose to cover X% of a target volume; GTV - X mm: Gross tumor volume evenly reduced by X mm

Assuming that the dose increases inward from the GTV boundary is concentrically layered in the spherical GTVs (10-45 mm), the distances of the IIV surfaces corresponding to 80% and 50% of the GTV (*D*_80%_ and *D*_50%_) from the GTV boundary are shown in Figure 1A. The GTV coverage values by the GTV - 2 mm and GTV - 4 mm are shown in Figure 1B. The isodose surfaces (IDS) of the GTV *D*_80%_ were 0.4-1.6 mm (<2 mm) inside the GTV boundary, while those of the GTV *D*_50%_ from the GTV boundary ranged from 1.0 mm to 4.6 mm (Figure 1A). Meanwhile, the GTV coverage values of the volumes of GTV evenly reduced by 2 mm and 4 mm increased from 21.6% to 75.6% and from 0.8% to 55.6%, respectively, as the GTV increased (Figure 1B).

**Figure 2:**
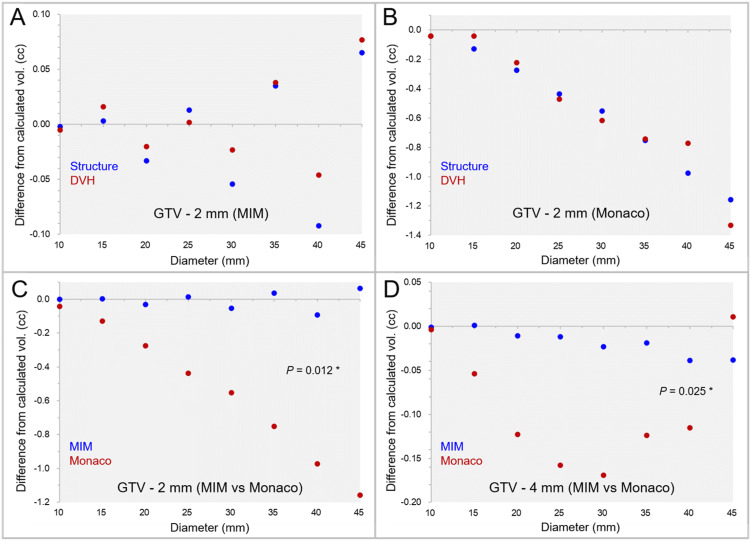
Comparison of functions to reduce volume evenly in two planning systems using spherical model volumes. The scatter plots show the differences between the GTV - 2 mm structures by MIM Maestro from the calculated volumes (A); the differences between the GTV - 2 mm structures by Monaco from the calculated volumes (B); the differences of the GTV - 2 mm structures between the two systems (C); and the differences of the GTV - 4 mm structures between the two systems (D). Further volume variations in the DVHs are also shown (A-B). The results of the WSRT are added (C-D). The horizontal axes show the diameter of sphere volumes, and the vertical axes show the differences of the structure volumes from the calculated values (A-D). vol: Volume; MIM: MIM Maestro; GTV: Gross tumor volume; DVHs: Dose-volume histograms; WSRT: Wilcoxon signed-rank test; GTV - X mm: Gross tumor volume evenly reduced by X mm.

The differences between the GTV - 2 mm and GTV - 4 mm volumes generated by the two systems from the calculated values are shown in Figures 2A-2D, along with further volume variations in the DVHs.

**Figure 3:**
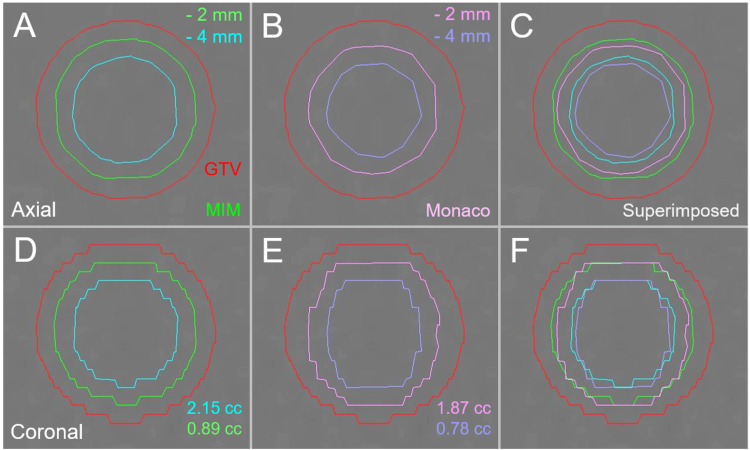
The differences in functions to reduce volume evenly between two planning systems in a 20-mm sphere model. The images show the target contours superimposed onto non-contrast-enhanced (CT) images (A-F); axial images (A-C); coronal images (D-F); by MIM Maestro (A and D); by Monaco (B and E); and the superimposed images (C and F). The differences in the volumes are equally reduced by 2 mm and 4 mm from the identical spherical model with a diameter of 20 mm. CT: Computed tomography; GTV: Gross tumor volume; GTV - X mm: Gross tumor volume evenly reduced by X mm; MIM: MIM Maestro

The differences of the GTV - 2 mm and GTV - 4 mm volumes from the calculated values were significantly larger in Monaco than in MIM Maestro. The example of the GTV - 2 mm and GTV - 4 mm structures generated by the two systems is shown in Figure 3.

The GTV - 2 mm and GTV - 4 mm structures in Monaco tended to be smaller than the original volume, and the unevenness of the contours was more noticeable in Monaco. Therefore, subsequent analyses were performed using the GTV - 2 mm and GTV - 4 mm structures generated by MIM Maestro.

**Figure 4:**
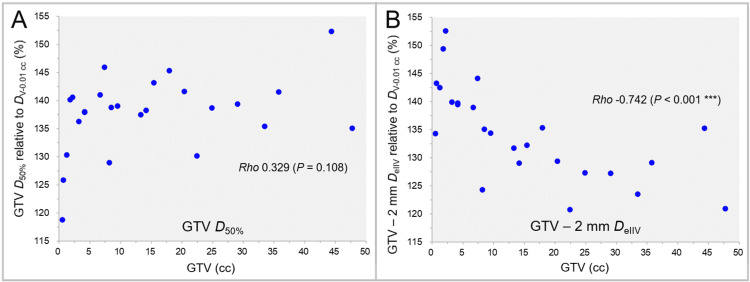
GTV D50% and GTV - 2 mm DeIIV as a function of GTV. The scatter plots show the correlations between the GTVs and the GTV *D*_50%_ relative to the GTV *D*_V-0.01 cc_ (A); and the GTV - 2 mm *D*_eIIV_ relative to the GTV *D*_V-0.01 cc_ (B). The results of SRCC are added. SRCC: Spearman’s rank correlation coefficient; GTV: Gross tumor volume; *D*_50%_: A minimum dose covering at least 50% of a target volume; *D*_V-0.01 cc_: A minimum dose to cover a target volume minus 0.01 cc; GTV - 2 mm: Gross tumor volume evenly reduced by 2 mm; *D*_eIIV_: A minimum dose to cover the irradiated isodose volume equivalent to a target volume on the dose-volume histogram

The GTV median dose (*D*_50%_) and the dose 2 mm inside the GTV boundary (GTV - 2 mm *D*_eIIV_), relative to the GTV *D*_V-0.01 cc_ (100%), as a function of GTV are shown in Figure 4.

There was no significant difference between the GTV *D*_50%_ and the GTV - 2 mm *D*_eIIV_ (WSRT, p = 0.109). Although no significant correlation was observed between the GTV and the GTV *D*_50%_ (Figure 4A), the GTV - 2 mm *D*_eIIV_ decreased significantly as the GTV increased (Figure 4B).

**Figure 5:**
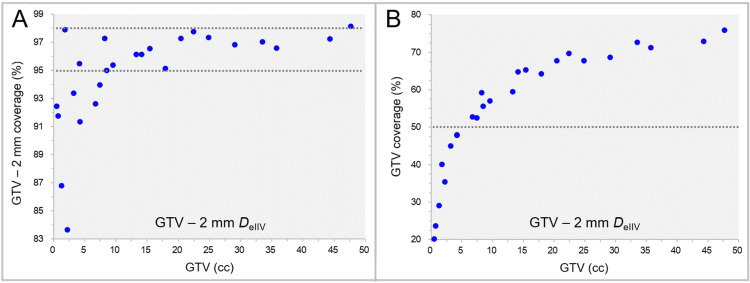
Coverage values of GTV - 2 mm and GTV by GTV - 2 mm DeIIV. The scatter plots show the correlations between the GTVs and the GTV - 2 mm coverage values by the GTV - 2 mm *D*_eIIV_ (A); and the GTV coverage values by the GTV - 2 mm *D*_eIIV_ (B). GTV: Gross tumor volume; GTV - 2 mm: Gross tumor volume evenly reduced by 2 mm; *D*_eIIV_: A minimum dose to cover the irradiated isodose volume equivalent to a target volume on the dose-volume histogram

The coverage values of the GTV - 2 mm and the GTV by the GTV - 2 mm *D*_eIIV_ are shown in Figure 5.

The coverage values of the GTV - 2 mm by the GTV - 2 mm *D*_eIIV_ were within the range of 95-98% in 68% of the cases, while those were <95% in 28% of the cases. The GTV coverage with the GTV - 2 mm *D*_eIIV_ ranged from 20.2% to 75.9%, in which those were within the range of 50-80% in 80% of the cases.

**Figure 6:**
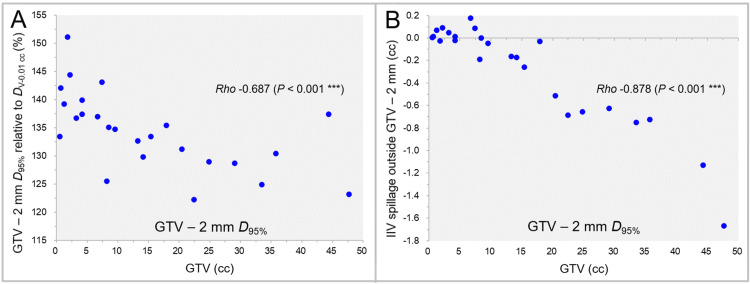
GTV - 2 mm D95% and the irradiated isodose volume spillage over GTV - 2 mm as a function of GTV. The scatter plots show the correlations between the GTVs and the GTV - 2 mm *D*_95%_ relative to the GTV *D*_V-0.01 cc_ (A); and the IIVs minus the GTV - 2 mm for the GTV - 2 mm *D*_95%_ (B). The results of SRCC are added. GTV: Gross tumor volume; GTV - 2 mm: Gross tumor volume evenly reduced by 2 mm; *D*_95%_: A minimum dose covering at least 95% of a target volume; *D*_V-0.01 cc_: A minimum dose to cover a target volume minus 0.01 cc; IIV: Irradiated isodose volume; SRCC: Spearman’s rank correlation coefficient.

The GTV - 2 mm *D*_95%_ relative to the GTV *D*_V-0.01 cc_ (100%) and the excess or deficiency of the IIV relative to the GTV - 2 mm volumes are shown in Figure 6 as a function of GTV.

The GTV - 2 mm *D*_95%_ decreased significantly as the GTV increased (Figure 6A), and the correlation between the *D*_eIIV_ and *D*_95%_ of the GTV - 2 mm was high (SRCC: rho = 0.983, p < 0.001***). However, there was a considerable excess or deficiency in the IIV of the GTV - 2 mm *D*_95%_ for the GTV - 2 mm (Figure 6B).

**Figure 7:**
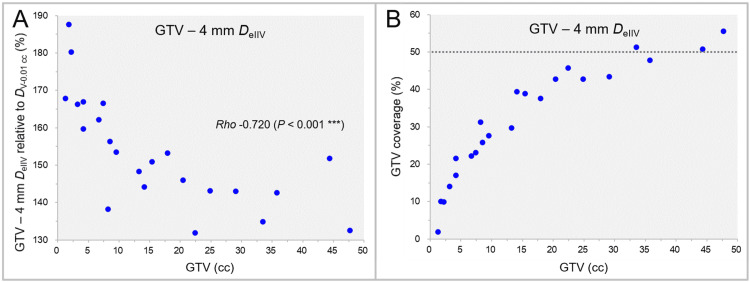
GTV - 4 mm DeIIV as a function of GTV and GTV coverage value by GTV - 4 mm DeIIV. The scatter plots show the correlations between the GTVs and the GTV - 4 mm *D*_eIIV_ relative to the GTV *D*_V-0.01 cc_ (A); and the GTV coverage values by the GTV - 4 mm *D*_eIIV_ (B). The result of SRCC is added in A. GTV: Gross tumor volume; GTV - 4 mm: Gross tumor volume evenly reduced by 4 mm; *D*_eIIV_: A minimum dose to cover the irradiated isodose volume equivalent to a target volume on the dose-volume histogram; *D*_V-0.01 cc_: A minimum dose to cover a target volume minus 0.01 cc; SRCC: Spearman’s rank correlation coefficient

The dose 4 mm inside the GTV boundary (GTV - 4 mm *D*_eIIV_), relative to the GTV *D*_V-0.01 cc_ (100%), and the GTV coverage value as a function of GTV (≥1.26 cc, 23 cases) are shown in Figure 7.

The GTV - 4 mm *D*_eIIV_ decreased significantly as the GTV increased (Figure 7A). The GTV coverage with the GTV - 4 mm *D*_eIIV_ ranged from 1.9% to 55.6%, which those were <50% in 87% of the cases (Figure 7B). The GTV - 4 mm *D*_eIIV_ was significantly higher than the GTV *D*_50%_ (WSRT, p < 0.001***).

**Figure 8:**
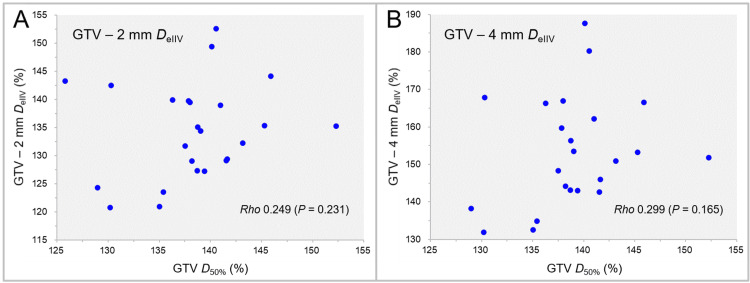
Correlations between GTV D50% and DeIIV of GTV - 2 mm and GTV - 4 mm. The scatter plots show the correlations between the GTV *D*_50%_ relative to the GTV *D*_V-0.01 cc_ and the GTV - 2 mm *D*_eIIV_ relative to the GTV *D*_V-0.01 cc_ (A); and the GTV - 4 mm *D*_eIIV_ relative to the GTV *D*_V-0.01 cc_ (B). The results of SRCC are added in A and B. GTV: Gross tumor volume; GTV - X mm: Gross tumor volume evenly reduced by X mm; *D*_eIIV_: A minimum dose to cover the irradiated isodose volume equivalent to a target volume on the dose-volume histogram; *D*_50%_: A minimum dose covering at least 50% of a target volume; *D*_V-0.01 cc_: A minimum dose to cover a target volume minus 0.01 cc; SRCC: Spearman’s rank correlation coefficient

The correlations between the GTV *D*_50%_ and the *D*_eIIV_s of the GTV - 2 mm and GTV - 4 mm are shown in Figure 8.

No significant correlation was observed between the GTV *D*_50%_ and the *D*_eIIV_s of the GTV - 2 mm (Figure 8A) and GTV - 4 mm (Figure 8B).

**Figure 9:**
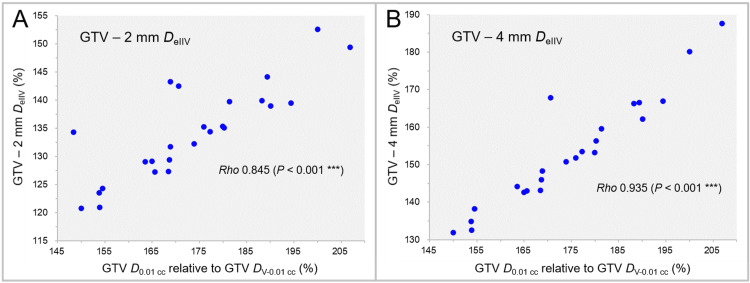
Correlations between D0.01 cc and DeIIVs of GTV - 2 mm and GTV - 4 mm. The scatter plots show the correlations between the GTV *D*_0.01 cc_ relative to the GTV *D*_V-0.01 cc_ and the GTV - 2 mm *D*_eIIV_ relative to the GTV *D*_V-0.01 cc_ (A); and the GTV - 2 mm *D*_eIIV_ relative to the GTV *D*_V-0.01 cc_ (B). The results of SRCC are added in A and B. GTV: Gross tumor volume; GTV - X mm: Gross tumor volume evenly reduced by X mm; *D*_eIIV_: A minimum dose to cover the irradiated isodose volume equivalent to a target volume on the dose-volume histogram; *D*_0.01 cc_: A minimum dose covering 0.01 cc of a target volume as the near-maximum dose; *D*_V-0.01 cc_: A minimum dose to cover a target volume minus 0.01 cc; SRCC: Spearman’s rank correlation coefficient

The correlations between the near-maximum dose of the GTV (*D*_0.01 cc_) and the *D*_eIIV_s of the GTV - 2 mm and GTV - 4 mm are shown in Figure 9.

The *D*_eIIV_s of the GTV - 2 mm (Figure 9A) and GTV - 4 mm (Figure 9B) increased significantly as the GTV *D*_0.01 cc_ increased. The correlation between the *D*_eIIV_ and the GTV *D*_0.01 cc_ was higher in the GTV - 4 mm than in the GTV - 2 mm.

## Discussion

An extremely inhomogeneous GTV dose, especially a concentrically-layered steep dose increase inside a GTV boundary, has both pros and cons. In SRS with ≥5 fractions for large BM with massive surrounding edema, tumor shape changes, including shrinking or enlargement, and/or its displacement can occur during irradiation [23-25]. If a GTV internal dose is not extremely inhomogeneous, e.g., 80% isodose coverage, tumor shrinkage and enlargement can occur with approximately equal probability [26-29]. In our experience, however, an extremely inhomogeneous GTV dose with a steep dose increase inside the GTV boundary tends to contribute to considerable tumor shrinkage during irradiation in many cases with the exception of BMs from colon and renal cell cancer [24,25,30]. Although significant tumor shrinkage during irradiation can contribute to early alleviation of the relevant symptoms, it may also increase high-dose exposures to the surrounding brain, leading to the development of symptomatic brain radionecrosis [24]. In a case with significant tumor shrinkage during multi-fraction SRS, the dose within 2-4 mm inside the GTV surface may have a greater impact on therapeutic efficacy and adverse effects than the GTV median dose (*D*_50%_) or near-maximum dose (*D*_2%_ or *D*_0.01 cc_).

In VMA-based SRS with the optimization that prioritizes the steepness of dose gradient and with dose prescription of a constant BED_10_ at the GTV boundary, the BED_10_ 2-4 mm inside the GTV boundary definitely decreases as the GTV increases. Furthermore, the internal BED_10_ decreases as the number of dose fractions increases to ensure safety for larger GTVs (Tables 1-2). The internal BED_10_ decline in large tumors can attenuate excessive dose exposures to the surrounding brain due to the GTV shrinkage during SRS with ≥5 fractions. However, when increasing the number of dose fractions for large tumors to ensure safety, the optimization that further increases the dose gradient inside the GTV boundary may be considered to enhance therapeutic efficacy.

In many facilities implementing linac-based SRS, dose prescription to a margin-added planning target volume boundary is common, in which the differences in marginal doses between the PTV and GTV are substantially different [5,20]. Therefore, reference to the GTV marginal dose is essential. Although *D*_98%_, *D*_50%_, and *D*_2%_ of a GTV may be optimal as the representative metrics to specify the major features of the DVH curve [16], other dosimetric parameters seem to be more relevant to maximal response and local control than these metrics. The GTV *D*_V-0.01 cc_ and *D*_0.01 cc_ instead of the *D*_98%_ and *D*_2%_ may be more appropriate as the near-minimum and near-maximum doses for a GTV of >0.20 cc, respectively (*D*_95%_ and *D*_5%_ for GTV ≤0.20 cc) [20]. In addition, the GTV *D*_50%_ has poor correlations with the doses 2 mm and 4 mm inside the GTV boundary, while the GTV *D*_80%_ is too close to the GTV boundary with the irradiated isodose surface less than 1-2 mm inside the boundary. The dose within a certain distance from the GTV boundary can be an independent evaluation metric of the GTV internal dose and may be more relevant to maximal response and local control for SRS of BMs. However, given the substantial variability in the volume several millimeters inside a GTV among planning systems, the accuracy of GTV - 2 mm or 4 mm volume should be examined in advance in the system used. The *D*_eIIV_, instead of the minimum dose with a fixed percentage coverage (e.g. *D*_98%_) is suitable for reporting the dose 2 mm and 4 mm inside the GTV boundary in terms of avoiding the over- or under-coverage [20,22]. Furthermore, a high coverage value of *D*_eIIV_ can be an indicator of the dose convergence inside a GTV surface. However, even with extremely inhomogeneous GTV doses, the *D*_eIIV_ may be unsuitable as the evaluation metric for the doses 2-4 mm inside the GTV boundary, when the dose increase inside the GTV boundary is not concentrically layered, and the high dose area does not converge to the GTV center while being dispersed. In such cases, evaluation with the *D*_95%_ of the GTV - 2 mm or 4 mm is likely appropriate instead of the *D*_eIIV_. Taken together, the importance of GTV dose metrics relevant to the maximal tumor response may be the GTV *D*_V-0.01 cc_, GTV - 2 mm *D*_eIIV_, GTV - 4 mm *D*_eIIV_, GTV *D*_50%_, and GTV *D*_0.01 cc_ in order. However, evaluation with the GTV - 4 mm *D*_eIIV_ is unsuitable for a GTV of ≤11-12 mm as the IIV of the GTV - 4 mm *D*_eIIV_ approximates the GTV *D*_0.01 cc_. Thus, supplemental evaluation with the GTV - 4 mm *D*_eIIV_ may be meaningful for a GTV of >2 cm.

This planning study was based on the results of a certain optimization method using VMA and has inherent limitations. Whether the *D*_eIIV_ of GTV - 2 mm or GTV - 4 mm is clinically more relevant to maximal response and local control than the GTV *D*_50%_ warrants further investigation. It is also necessary to clarify the significance of concentrically-layered steep dose increase inside a GTV boundary in maximal tumor response and to demonstrate a GTV central or maximum dose that is more than twice the GTV marginal dose being generally harmless.

## Conclusions

A dose of 2 mm or 4 mm inside a GTV surface has a low correlation with the GTV *D*_50%_ or other minimum dose with a fixed coverage. The dose within a certain distance from the GTV boundary can be an independent evaluation metric of the GTV internal dose. The *D*_eIIV_ instead of the minimum dose of a fixed percentage coverage (e.g. *D*_98%_) is suitable as the representative index in terms of avoiding over- or under-coverage. The *D*_eIIV_ of GTV - 2 mm and GTV - 4 mm may be more relevant to maximal response and local control for SRS of BM, in which the high coverage value can reflect high dose convergence inside the GTV. In VMA-based SRS with a steep dose gradient, the doses 2-4 mm inside a GTV decrease significantly as the GTV increases, which can attenuate excessive dose exposures to the surrounding brain due to the GTV shrinkage during SRS with ≥5 fractions.
